# Rapid, accurate, computational discovery of Rho-independent transcription terminators illuminates their relationship to DNA uptake

**DOI:** 10.1186/gb-2007-8-2-r22

**Published:** 2007-02-21

**Authors:** Carleton L Kingsford, Kunmi Ayanbule, Steven L Salzberg

**Affiliations:** 1 Center for Bioinformatics and Computational Biology, University of Maryland, College Park, MD 20742, USA

## Abstract

**Background:**

In many prokaryotes, transcription of DNA to RNA is terminated by a thymine-rich stretch of DNA following a hairpin loop. Detecting such Rho-independent transcription terminators can shed light on the organization of bacterial genomes and can improve genome annotation. Previous computational methods to predict Rho-independent terminators have been slow or limited in the organisms they consider.

**Results:**

We describe TransTermHP, a new computational method to rapidly and accurately detect Rho-independent transcription terminators. We predict the locations of terminators in 343 prokaryotic genomes, representing the largest collection of predictions available. In *Bacillus subtilis*, we can detect 93% of known terminators with a false positive rate of just 6%, comparable to the best-known methods. Outside the Firmicutes division, we find that Rho-independent termination plays a large role in the *Neisseria *and *Vibrio *genera, the Pasteurellaceae (including the *Haemophilus *genus) and several other species. In *Neisseria *and Pasteurellaceae, terminator hairpins are frequently formed by closely spaced, complementary instances of exogenous DNA uptake signal sequences. We quantify the propensity for terminators to include these sequences. In the process, we provide the first discussion of potential uptake signals in *Haemophilus ducreyi *and *Mannheimia succiniciproducens*, and we discuss the preference for a particular configuration of uptake signal sequences within terminators.

**Conclusion:**

Our new fast and accurate method for detecting transcription terminators has allowed us to identify and analyze terminators in many new genomes and to identify DNA uptake signal sequences in several species where they have not been previously reported. Our software and predictions are freely available.

## Abstract 2

Using a novel computational method, an extensive collection of predicted Rho-independent transcription terminators is derived from 343 prokaryotes, offering insight into their relationship to DNA uptake

## Background

Rho-independent (also known as intrinsic) terminators are sequence motifs found in many prokaryotes that cause the transcription of DNA to RNA to stop. These termination signals typically consist of a short, often GC-rich hairpin followed by a sequence enriched in thymine residues [1]. The importance of Rho-independent termination varies across bacteria. In some bacteria, such as *Escherichia coli*, a large fraction of termination is mediated by the Rho protein or its homologs (reviewed in [2]). In others, such as *Bacillus subtilis*, Rho homologs play a smaller role, and Rho-independent termination is the norm.

Detection of transcription termination sites is key to understanding the operon structure of bacterial genomes. Understanding the operons, in turn, gives us strong hints about gene function. Computational detection of termination signals is the only practical means of identifying large numbers of terminators today, and few experimentally verified terminators exist outside of *B. subtilis *and *E. coli*. Several previous computational methods [3,4] have relied on simple decision boundaries to separate terminators from non-terminators after training on experimentally known terminating and non-terminating sequences. Other studies have considered only the hairpin portion of potential terminators [5,6]. Due to lack of sequence data, previous systems (for example, [4,7]) have tended to focus on *E. coli *or on only a portion of the now-available genomes.

We describe here TransTermHP, a computational method for the rapid and accurate detection of these signals in genomic DNA. TransTermHP searches genomic DNA for terminators and assigns each candidate terminator a score related to the likelihood that it arose by chance. We assess sensitivity and specificity of our predictions using a set of experimentally verified operons [3]. Our method achieves accuracy comparable with a recent method [3] while at the same time being much faster and not dependent on a training set of known terminators.

A previous system [8] by one of the authors of the current study predicted terminators within intergenic regions by contrasting candidates with terminator-like structures that occur within genes. Since the intragenic sequences were used as a background signal, the system could not assign scores to structures inside genes and its scores depended on the distribution of relatively rare terminator-like sequences inside genes. TransTermHP uses a completely new, more general model of background signal and a new scoring function that makes it less sensitive to the accuracy of the genome annotation. TransTermHP is built around a new search algorithm that employs dynamic programming to search genomes ten times faster than the system of Ermolaeva *et al*. [8] and hundreds of times faster than the method of de Hoon *et al*. [3]. The algorithm can scan a 4 megabase genome in under 50 seconds on a single commodity processor.

Using the accuracy and speed of the new method we have made predictions of Rho-independent terminators for 343 bacterial and archaeal genomes (comprising the complete collection of finished prokaryotic genomes available from GenBank). These predictions are available on the web and represent the largest and most varied collection of putative terminators available to date.

A recent paper [3] considered Rho-independent terminators in the Firmicutes division of bacteria extensively and found within them a large number of intrinsic termination signals, a finding we corroborate. Outside the Firmicutes, we find that 15 organisms (from the *Neisseria*, *Vibrio*, and *Psychrobacter *genera, the Pasteurellaceae, as well as *Pseudoalteromonas haloplanktis*, *Desulfovibrio desulfuricans*, and *Fusobacterium nucleatum*) also appear to employ intrinsic termination for a large fraction of their termination signals.

It has been noted [9-11] for some organisms in the *Neisseria *genus and the Pasteurellaceae that transcription terminators often include DNA uptake signal sequences (USSs). These uptake signals are short (approximately 9-11 nucleotides (nt)), highly conserved sequences that occur repeatedly (generally ≥ 1,000 times) in a bacterial genome and that facilitate the incorporation of exogenous DNA into the cells of naturally transformable bacteria (see [12,13] for reviews). Many of the termination signals predicted by TransTermHP in these organisms include USS motifs within their hairpins. We expand past analysis of this phenomenon to additional organisms and present evidence that this is not an artifact of the prediction method nor a result of some other preference for a hairpin configuration of USS sites: these organisms appear to have co-opted the uptake signals for use in transcription termination. In addition, we propose a new USS motif for *H. ducreyi *that has been overlooked by previous studies. Finally, we discuss a bias in the configuration of USS motifs that form hairpins.

## Results and discussion

### Algorithm to search for candidate terminators

**Figure 1:**
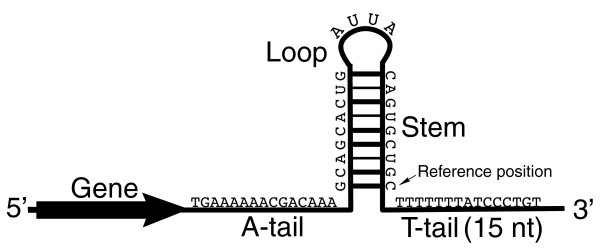
Schematic of the terminator motif for which TransTermHP searches. The terminators consist of a short stem-loop hairpin followed by a thymine-rich region on their 3' side. For the results reported here, TransTermHP was restricted to find terminators for which each side of the stem is ≥ 4 nt, the length of the loop is ≥ 3 nt and ≤ 13 nt, and the total length of the stem-loop was ≤ 59 nt.

TransTermHP searches whole prokaryotic genomes for intrinsic terminators of the type depicted in Figure 1: a short, low-energy hairpin followed downstream by a stretch of thymine nucleotides (which are transcribed into uracils). The 15 bases on the 3' end of the motif are called the 'T-tail', while the 15 bases on the 5' side are called the 'A-tail', as the same sequence often functions as a terminator on both strands, requiring the preservation of adenines at the 5' end (which are the thymine residues of the T-tail at the 3' end on the opposite strand). Due to the difference in stability of a G-U pairing versus the complementary C-A pairing in RNA, the hairpins found on one strand of the genome will, in general, be different from those found on the opposite strand. Consequently, the search algorithm described below is performed once for each strand.

Every window of DNA of length six that contains at least three thymines is examined to see whether it is the hairpin-proximal end of a tail of a potential terminator. The position adjacent to the 5' side of such a window is a candidate 'reference position' for the 3' end of a terminator hairpin and the sequence downstream of this position is the potential tail. The first 15 bases of the potential tail sequence are scored using a heuristic function from d'Aubenton Carafa *et al*. [4], which places more weight on thymines near the hairpin:

tail_score(s)=−∑n=115xn     (1)
 MathType@MTEF@5@5@+=feaafiart1ev1aaatCvAUfeBSjuyZL2yd9gzLbvyNv2Caerbhv2BYDwAHbqedmvETj2BSbqee0evGueE0jxyaibaiKI8=vI8tuQ8FMI8Gi=hEeeu0xXdbba9frFj0=OqFfea0dXdd9vqai=hGuQ8kuc9pgc9s8qqaq=dirpe0xb9q8qiLsFr0=vr0=vr0dc8meaabaqaciGacaGaaeqabaqadeqadaaakeaaieaacaWF0bGaa8xyaiaa=LgacaWFSbGaa83xaiaa=nhacaWFJbGaa83Baiaa=jhacaWFLbGaa8hkaiaa=nhacaWFPaGaeyypa0JaeyOeI0YaaabCaeaacaWG4bWaaSbaaSqaaiaad6gaaeqaaaqaaiaad6gacqGH9aqpcaaIXaaabaGaaGymaiaaiwdaa0GaeyyeIuoakiaaxMaacaWLjaWaaeWaaeaacaaIXaaacaGLOaGaayzkaaaaaa@4CB2@

where

xn={0.9xn−1if the nth nucleotide is T0.6xn−1otherwise
 MathType@MTEF@5@5@+=feaafiart1ev1aaatCvAUfeBSjuyZL2yd9gzLbvyNv2Caerbhv2BYDwAHbqedmvETj2BSbqee0evGueE0jxyaibaiKI8=vI8tuQ8FMI8Gi=hEeeu0xXdbba9frFj0=OqFfea0dXdd9vqai=hGuQ8kuc9pgc9s8qqaq=dirpe0xb9q8qiLsFr0=vr0=vr0dc8meaabaqaciGacaGaaeqabaqadeqadaaakeaacaWG4bWaaSbaaSqaaiaab6gaaeqaaOGaeyypa0ZaaiqabeaafaqaaeGacaaabaGaaGimaiaac6cacaaI5aGaamiEamaaBaaaleaacaqGUbGaeyOeI0IaaGymaaqabaaakeaacaqGPbGaaeOzaiaabccacaqG0bGaaeiAaiaabwgacaqGGaGaamOBaiaabshacaqGObGaaeiiaiaab6gacaqG1bGaae4yaiaabYgacaqGLbGaae4BaiaabshacaqGPbGaaeizaiaabwgacaqGGaGaaeyAaiaabohacaqGGaGaaeivaaqaaiaaicdacaGGUaGaaGOnaiaadIhadaWgaaWcbaGaaeOBaiabgkHiTiaaigdaaeqaaaGcbaGaae4BaiaabshacaqGObGaaeyzaiaabkhacaqG3bGaaeyAaiaabohacaqGLbaaaaGaay5Eaaaaaa@628F@

for *n *= 1...15 and *x*_0 _= 1.

The energy of potential hairpin configurations adjacent to a reference position can be found efficiently with a dynamic programming algorithm similar to classic RNA folding algorithms [14,15]. The recurrence equation for this algorithm is given below for completeness. The table entry **hairpin_score**[*i*,*j*] gives the cost of the best hairpin structure for which the base of the 5' stem is at nucleotide position *i *and the base of the 3' stem is at position *j*. The entry **hairpin_score**[*i*,*j*] can be computed recursively as follows:

hairpin_score[i,j]=min⁡{loop_pen(j−i+1)loopenergy(i,j)+hairpin_score[i+1,j−1]match or mismatchgap+hairpin_score[i+1,j]gap on left stemgap+hairpin_score[i,j−1]gap on right stem     (2)
 MathType@MTEF@5@5@+=feaafiart1ev1aaatCvAUfeBSjuyZL2yd9gzLbvyNv2Caerbhv2BYDwAHbqedmvETj2BSbqee0evGueE0jxyaibaiKI8=vI8tuQ8FMI8Gi=hEeeu0xXdbba9frFj0=OqFfea0dXdd9vqai=hGuQ8kuc9pgc9s8qqaq=dirpe0xb9q8qiLsFr0=vr0=vr0dc8meaabaqaciGacaGaaeqabaqadeqadaaakeaacaqGObGaaeyyaiaabMgacaqGYbGaaeiCaiaabMgacaqGUbGaae4xaiaabohacaqGJbGaae4BaiaabkhacaqGLbGaai4waiaadMgacaGGSaGaamOAaiaac2facqGH9aqpciGGTbGaaiyAaiaac6gadaGabeqaauaabaqaeiaaaaqaaGqabiaa=XgacaWFVbGaa83Baiaa=bhacaqGFbGaa8hCaiaa=vgacaWFUbGaaiikaiaadQgacqGHsislcaWGPbGaey4kaSIaaGymaiaacMcaaeaacaqGSbGaae4Baiaab+gacaqGWbaabaGaa8xzaiaa=5gacaWFLbGaa8NCaiaa=DgacaWF5bGaaiikaiaadMgacaGGSaGaamOAaiaacMcacqGHRaWkcaWFObGaa8xyaiaa=LgacaWFYbGaa8hCaiaa=LgacaWFUbGaae4xaiaa=nhacaWFJbGaa83Baiaa=jhacaWFLbGaai4waiaadMgacqGHRaWkcaaIXaGaaiilaiaadQgacqGHsislcaaIXaGaaiyxaaqaaiaab2gacaqGHbGaaeiDaiaabogacaqGObGaaeiiaiaab+gacaqGYbGaaeiiaiaab2gacaqGPbGaae4Caiaab2gacaqGHbGaaeiDaiaabogacaqGObaabaGaam4zaiaadggacaWGWbGaey4kaSIaa8hAaiaa=fgacaWFPbGaa8NCaiaa=bhacaWFPbGaa8NBaiaab+facaWFZbGaa83yaiaa=9gacaWFYbGaa8xzaiaacUfacaWGPbGaey4kaSIaaGymaiaacYcacaWGQbGaaiyxaaqaaiaabEgacaqGHbGaaeiCaiaabccacaqGVbGaaeOBaiaabccacaqGSbGaaeyzaiaabAgacaqG0bGaaeiiaiaabohacaqG0bGaaeyzaiaab2gaaeaacaWGNbGaamyyaiaadchacqGHRaWkcaWFObGaa8xyaiaa=LgacaWFYbGaa8hCaiaa=LgacaWFUbGaae4xaiaa=nhacaWFJbGaa83Baiaa=jhacaWFLbGaai4waiaadMgacaGGSaGaamOAaiabgkHiTiaaigdacaGGDbaabaGaae4zaiaabggacaqGWbGaaeiiaiaab+gacaqGUbGaaeiiaiaabkhacaqGPbGaae4zaiaabIgacaqG0bGaaeiiaiaabohacaqG0bGaaeyzaiaab2gaaaaacaGL7baacaWLjaGaaCzcamaabmaabaGaaGOmaaGaayjkaiaawMcaaaaa@D247@

The function **energy**(*i*,*j*) gives the cost of pairing the nucleotide at *i *with that at *j*, and **loop_pen**(*n*) gives the cost of a hairpin loop of length *n*. The hairpin's loop is forced to have a length between 3 and 13 nt, inclusive, by setting **loop_pen**(*n*) to a large constant for any *n *outside that range. The constant 'gap' gives the cost of not pairing a base with some base on the opposite stem and thus introducing a gap on one side of the hairpin stem. The values for these costs for evaluating the quality of hairpins (Table 1) are taken from Ermolaeva *et al*. [8], where they were trained from a set of 70 previously known *E. coli *terminators [4]. The selection of these values are the only instance of training, and the performance of TransTermHP is robust against other reasonable choices of the parameters.

Since terminators are small structures, we are only interested in values of **hairpin_score**[*i*,*j*] for which *j *- *i *+ 1 is a small constant. Hence, we need to keep only a small portion of the table and thus the space used is constant relative to the size of the genome. Because we fill at most a constant-sized table for each nucleotide position the running time is asymptotically linear in the number of candidate reference positions. For the predictions below, we require the total extent of the stem-loop structure to be less than 59 nt long. If *j *is a candidate reference position then *j *+ 1 is likely to be one as well. By appropriately arranging the values of the **hairpin_score**[*i*,*j*] table in memory, we can reuse the values computed for index *j *when considering index *j *+ 1, resulting in a further practical increase in speed. We refer the reader to the available source code for implementation details (Additional data file 1).

The computed **hairpin_score**[*i*,*j*] gives the energy of the best hairpin with endpoints *i *and *j*. Fixing *j *at the reference position, we try all possible 5' endpoints *i *to find the extent of the best hairpin adjacent to the reference position *j*. In other words, the 5' endpoint is taken to be argmin_*i *_**hairpin_score**[*i*,*j*] where *i *ranges over the possible endpoints within a bounded distance of *j*. To reduce the number of candidates that we must store, we keep only candidates with tail score ≤ -2.5 and hairpin scores ≤ -2. In addition, we discard any candidates with stems of length ≤ 4.

The above search procedure will find many terminators that overlap. While some overlapping terminators may be of interest (as candidates for terminator/antiterminator pairs, for example), others clearly are not. In particular, we remove from consideration any terminator that is a subsequence of another terminator with better hairpin and tail scores. Because the hairpins of bidirectional terminators tend to be flanked by stretches of adenines on one side and complementary thymines on the other, we also remove any terminator for which the bottom half of the stem appears to be a hybridization between the two tails of a bidirectional terminator. Specifically, we discard any terminator that is a super-sequence of another terminator and has five or more consecutive adenines in the 5'-most half of the 5' side of the hairpin stem and five or more consecutive thymines in the 3'-most half of the 5' side of the hairpin stem. To further discourage the A-tail and T-tail regions of potential bidirectional terminators from pairing, we require that the first base in the hairpin base closest to the T-tail not be a thymine.

In practice, our search procedure is very fast. It can search the complete 4.2 megabases of the *B. subtilis *genome (including intragenic regions) using a standard desktop machine in under one minute. In contrast, on the same machine, a recent method [3] took over two days to scan only the regions immediately downstream of genes. The speed of our search algorithm facilitates interactive experimentation and refinement and permits predictions for many genomes to be undertaken. Our specialized, dynamic-programming-based hairpin search algorithm is the basis for TransTermHP's speed. While this algorithm does not have the biophysical accuracy of more advanced RNA folding programs (for example, [16,17]), such accuracy is likely unnecessary for prediction of the short, generally high-quality hairpins that constitute transcription terminators.

### Function to evaluate the quality of terminators

The search process assigns a hairpin score *H *and a tail score *T *to every potential terminator. From these values and genomic context we compute a combined score as a measure of the overall quality of the putative terminator. Previous methods [3,4] have distilled a single score from the hairpin and tail scores by using a linear combination of the two values, where the weights in the linear combination are the result of a training process. Here, we take a different approach to avoid training with limited data. We assign a score *C*_*gc*_(*H*,*T*), defined below, that is related to the probability of finding a terminator of equal or better quality in a random sequence and is a measure of how unlikely the structure is to have arisen by chance.

Let *R*_*gc*_(*H*,*T*) be the number of terminators with hairpin score ≤ *H *and tail score ≤ *T *found in a random sequence of length *L *with GC content *gc*. We define the combined score as:

Cgc(H,T)=(−100/log⁡(L))log⁡(max⁡{1,Rgc(H,T)}L).     (3)
 MathType@MTEF@5@5@+=feaafiart1ev1aaatCvAUfeBSjuyZL2yd9gzLbvyNv2Caerbhv2BYDwAHbqedmvETj2BSbqee0evGueE0jxyaibaiKI8=vI8tuQ8FMI8Gi=hEeeu0xXdbba9frFj0=OqFfea0dXdd9vqai=hGuQ8kuc9pgc9s8qqaq=dirpe0xb9q8qiLsFr0=vr0=vr0dc8meaabaqaciGacaGaaeqabaqadeqadaaakeaacaWGdbWaaSbaaSqaaiaadEgacaWGJbaabeaakiaacIcacaWGibGaaiilaiaadsfacaGGPaGaeyypa0JaaiikaiabgkHiTiaaigdacaaIWaGaaGimaiaac+caciGGSbGaai4BaiaacEgacaGGOaGaamitaiaacMcacaGGPaGaciiBaiaac+gacaGGNbWaaeWaaeaadaWcaaqaaiGac2gacaGGHbGaaiiEaiaacUhacaaIXaGaaiilaiaadkfadaWgaaWcbaGaam4zaiaadogaaeqaaOGaaiikaiaadIeacaGGSaGaamivaiaacMcacaGG9baabaGaamitaaaaaiaawIcacaGLPaaacaGGUaGaaCzcaiaaxMaadaqadaqaaiaaiodaaiaawIcacaGLPaaaaaa@5B1B@

Thus, if many terminator-like structures in random sequences have both better tails and better hairpins than the terminator under consideration, the terminator will receive a low score. *R*_*gc*_(*H*,*T*) is computed empirically using randomly generated DNA sequences of length *L *= 20 Mb where each base is drawn from a distribution so that the generated sequence has expected GC content *gc*. The maximization in the numerator ensures that the expression remains well-defined even if no terminator-like structure with hairpin score ≤ *H *and tail score ≤ *T *is seen in the random sequence. The -100/log(*L*) factor normalizes the scores to range between 0 and 100. Ignoring this normalization, the combined score is the log of an estimate of the probability that a terminator as good or better would occur at a given position in a random sequence. In contrast to the scheme used in a predecessor system [8], this combined score allows assignment of scores to structures inside genes, is monotonically decreasing in each dimension, and smoothly handles candidates that occur in the real data less often than expected by chance.

The function *C*_*gc *_depends on the genome's GC content because the expected distribution of hairpin energies and the likelihood of a good T-tail vary with the frequency of G and C nucleotides. We use the empirically computed intra- or intergenic GC-bias for the value of *gc *in *C*_*gc *_depending on whether or not the putative terminator occurs inside an annotated coding region. To improve efficiency, *C*_*gc *_is calculated approximately from a set of pre-computed tables.

### Validation of predictions in *B. subtilis*

We ran TransTermHP on the complete genome of *B. subtilis*, an organism for which Rho-independent termination is suspected to play a predominant role. For each gene, we take the highest scoring terminator (according to equation 3) in the region beginning 25 nt upstream of its stop codon continuing until either the start of a gene on the same strand, or 500 nt downstream of the stop codon, whichever is shorter. In the case of terminators with tied scores, we choose the terminator closest to the stop codon of the upstream gene.

To determine the sensitivity and specificity of our search procedure and scoring scheme and to facilitate comparisons, we follow the testing methodology of de Hoon *et al*. [3], who provide a set of operons with experimental support [3] that they use to derive examples of terminating and non-terminating regions in *B. subtilis*. They take as positive examples those regions annotated in this data set as having terminators. No terminators should be found following genes that are internal to an experimentally validated operon and do not have read-through terminators following them. Any such region containing a predicted terminator is considered a false positive. Using the most recent GenBank annotation, this yields 458 positive and 562 negative examples (which differs slightly from the 463 positive and 567 negative regions reported by [3], due to differences in annotations).

**Figure 2:**
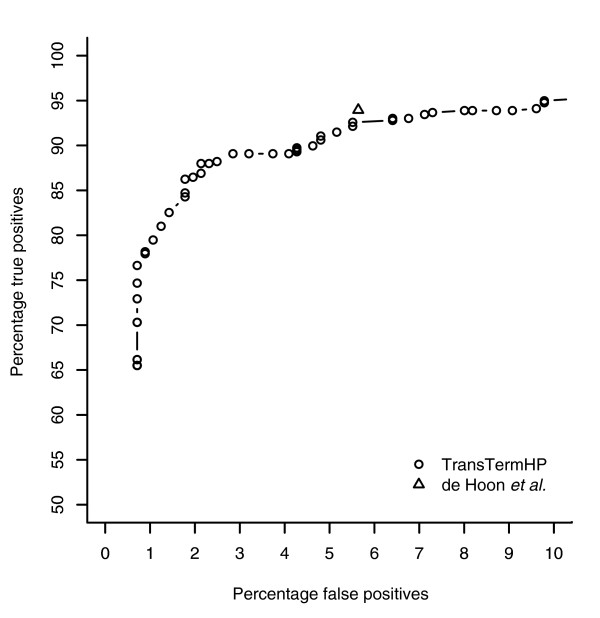
Performance of TransTermHP in *B. subtilis*. Receiver operator characteristic (ROC) curve showing the percentage of positive regions for which TransTermHP finds a terminator for various low false positive rates (circles), using a data set derived from experimentally verified operons [3]. The reported performance of the method described by de Hoon *et al*. [3] on this set after training is also shown (triangle). TransTermHP performs comparably without fitting parameters to the data set.

The percentage of regions where a terminator is experimentally expected for which TransTermHP finds a terminator is shown in Figure 2 for various false positive rates. When TransTermHP is set at the extremely conservative false positive rate of 0.7%, it finds a terminator in 77% of the positive regions, suggesting that there is a set of exceptionally good terminators for which prediction is easy. Sensitivity rapidly rises, reaching 88% by the time the false positive rate has increased to just 2.1%.

de Hoon *et al*. [3] introduced a method to predict terminators in which they learn a decision rule (line) separating terminators from non-terminators in the two-dimensional plane where one axis marks hairpin energy and the other measures the quality of the terminator tail. They report 93.95% sensitivity at 94.36% specificity after fitting the parameters of their decision rule to optimize performance on this data set. TransTermHP performs comparably with no real training: TransTermHP achieves 93.0% sensitivity at 93.6% specificity. The slightly higher numbers for the decision rule method may result from over-fitting, and its performance may not generalize outside of the training set. Indeed, the 13 terminators predicted by the decision rule method for regions in which TransTermHP finds no terminator at this specificity tend to have higher hairpin energies than other predictions, suggesting that their classification may be more affected by slight changes in the learned hyperplane. (The average reported hairpin energy of these 13 is -7.7 kcal/mol, while the average hairpin energy of all terminators reported by de Hoon *et al*. [3] in *B. subtilis *is -14 kcal/mol.)

It is interesting to note the apparent accuracy that TransTermHP achieves without significant training. One may have assumed that other palindromic sequences (such as certain classes of transcription factor binding sites) often found in intergenic regions would contribute to a high false positive rate. While some false positives may come from such signals, their number does not seem to impact performance significantly. This makes sense from a mechanistic viewpoint: whatever other functions such a sequence has, if it looks like a terminator, it will interrupt termination, and so such motifs will be selected against in regions in which termination is undesirable. While more advanced machine learning techniques may be helpful for improving performance, they do not seem essential for eliminating false positives.

At comparable false positive rates, TransTermHP and the decision rule method [3] make similar predictions following 87% of *B. subtilis *genes. For these genes, either both methods predict no terminator is present in the downstream region or the terminator predicted by de Hoon *et al*. [3] is contained within the terminator predicted by TransTermHP (including 15 flanking nucleotides on either side of the hairpin). For 3% of the genes, TransTermHP predicts a terminator where none is predicted by the decision rule method, and for 5.8% of genes each method predicts a different terminator. Hence, while the methods agree on a large core of terminator predictions, they also complement each other with differing predictions for terminators following 544 genes.

At a false positive rate of 10%, TransTermHP is unable to find any structures distinguishable from those found in random sequence in 5% of the positive regions, and for any false positive rate < 100% there remain 3.3% of the positive regions that do not contain a predicted terminator. It is possible that these regions are incorrectly labeled, rely on other methods of termination (for example, Rho-mediated), or have terminators outside of the search region (that is, far from the stop codon of the final gene of an operon). Alternatively, terminators in these regions may be functionally constrained to be weak terminators to enable occasional read-through, or it may be that there are structures within these regions that function well as terminators even though they cannot be distinguished from random sequences.

We label as 'high confidence' those terminators with a score greater than or equal to the cutoff necessary to achieve a 2.5% false positive rate in *B. subtilis *(yielding an 88% true positive rate). In the rest of this paper, we analyze high-confidence terminators that we predict in organisms without experimentally determined terminators.

### Terminator predictions for 343 organisms

We used TransTermHP to predict terminators for all the complete bacterial and archaeal genomes currently available from GenBank (343 genomes at the time of this study). Because of the greatly improved speed of TransTermHP, this requires less than four CPU hours. Complete predictions and the best terminator following each gene in each of the organisms are available online [18].

Due to a lack of large-scale experimental evidence, we are not able to assess the sensitivity/specificity tradeoff for TransTermHP on genomes other than that of *B. subtilis *as discussed above. However, as previously noted [3], those genes that are followed by at least two convergently transcribed genes (that is, → ← ←) are likely final genes in a transcription unit, and so we expect to find a termination signal situated downstream of them. We label these regions 'robust' tail-to-tail regions. We use the percentage of such regions in a genome that contain a high-confidence terminator as a statistic that measures both TransTermHP's sensitivity and the importance of Rho-indepenent termination in an organism. Unfortunately, we cannot untangle the two: the discovery of few terminators in these tail-to-tail regions may indicate a low importance of the hairpin/uracil-tail motif in termination, or it may indicate that the terminators are not sufficiently different from random sequences under our scoring scheme. Because of the accuracy of TransTermHP in *B. subtilis*, we would expect the former is true, but can not rule out the latter. The terminators predicted in robust tail-to-tail regions for each of the organisms are available in Additional data file 2.

The apparent importance of Rho-independent termination varies across the taxa of prokaryotes. A few previous studies considered sufficiently many organisms from a phylogenetic grouping to assess the relative importance of Rho-independent transcription termination for species in that grouping. TransTermHP's predictions are in line with these previous studies. Among our predictions, archaea generally do not contain high-confidence terminators: averaged over the 27 archaeal genomes, only 11% of the robust tail-to-tail regions contain them. This is in agreement with previous studies that showed that hairpins are not over-represented following genes in archaea [5,6]. That TransTermHP finds few terminators among these organisms is further evidence that it has a low false positive rate at the high-confidence threshold.

A recent study [3] found intrinsic termination to be the dominant termination mechanism among Firmicutes, a finding that our predictions confirm. Across the 56 Bacilli, on average 79% of the considered tail-to-tail regions contained a high-confidence terminator. Among the 7 Clostridia and 16 Mollicutes, fewer terminators were found (on average, 65% and 59%, respectively). Only 9 Firmicutes had high-confidence terminators in fewer than 60% of their robust tail-to-tail regions. Eight of these are Mollicutes; the ninth is the *Clostridium Moorella thermoacetica*. In agreement with [6], terminators were rarely found in *Mycoplasma genitalium *(20%) and *Mycoplasma pneumoniae *(24%). However, no Rho homolog is known to be present in these genomes [19], and so either a novel terminator mechanism is used, or the termination signals present in these organisms are weaker and more similar to random sequences.

**Figure 3:**
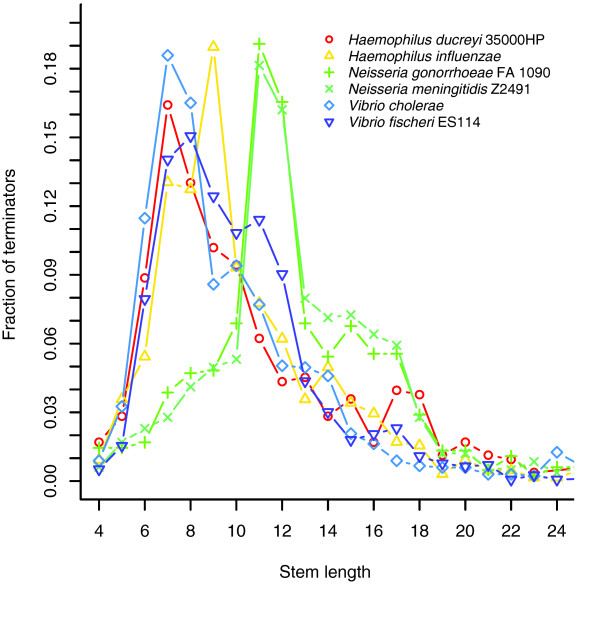
Stem lengths for high-confidence terminators in six organisms. These six organisms exhibit quite different distributions of stem lengths, with *Neisseria *having, on average, the longest stems, and the *H. ducreyi *and *V. cholerae *having the shortest. Because the statistics are computed using only high-confidence terminators, they may be skewed toward longer stem lengths.

In addition, several non-Firmicutes have a high-confidence terminator in a large fraction of their tail-to-tail regions (Table 2), suggesting that Rho-independent termination plays an important role. Organisms in the *Neisseria *and *Vibrio *genera, the Pasteurellaceae as well as several others listed in Table 2 employ Rho-independent termination extensively. All except *F. nucleatum *are proteobacteria. The distribution of stem lengths for the best, high-confidence terminators following genes for six of these organisms are shown in Figure 3, with the caveat that, because we focus on high-confidence terminators, these distributions may be skewed toward high-quality hairpins. The *Neisseria *genus has, among these, the longest stems on average. Their long stems, however, are accompanied by fewer thymines in their tail regions. These long stem lengths (mode = 11) are necessary to accommodate the highly conserved uptake sequence signals (see below) that are prevalent in these organisms.

### Relationship to DNA uptake

#### Prevalence of DNA uptake signals in terminators

Hundreds of copies of short, highly conserved DNA segments (called USSs) aid some bacteria such as *N. meningitidis *and *Haemophilus influenzae *in selectively incorporating homologous exogenous DNA [12,13,20-22]. These uptake signal sequences have been found to frequently occur within transcription terminator hairpins [9-11,22]. Smith *et al*. [11] examined *N. meningitidis*, *N. gonorrhoeae*, and *H. influenzae *and note that, on small scales, the distance between copies of the USS is not randomly distributed but rather there is an excess of copies of the motif on opposite strands within a small distance of one another, thus forming a sequence that may fold into a hairpin when transcribed into RNA.

We considered the highest-scoring, high-confidence terminator in the 500 nt downstream of each gene (beginning 25 nt upstream of the stop codon), if one exists. For several organisms in the *Neisseria *and Pasteurellaceae groupings, we found that a large percentage of these putative terminators contain the known USS motifs (Table 3). Here, we say a terminator contains the motif if the motif or its reverse complement is present in the sequence consisting of the hairpin and two flanking residues on each side. (Inclusion of the flanking residues is necessary because the Pasteurellacae motif begins with an AA dinucleotide and TransTermHP will not include pairings between the A-tail and T-tail in its reported hairpin.) In fact, approximately 55% of such terminators in the *Neisseria *contain an exact match to the known uptake signal sequence (GCCGTCTGAA) for that genus. Approximately 40% of genes are followed by a high-confidence terminator, suggesting that most operon ends contain a high-confidence terminator, and thus many operons end with a terminator that contains the USS motif.

Among Pasteurellaceae, the percentage of terminators containing known USSs is lower (between 17% and 29%). Though *P. multocida *has a larger genome than *H. influenzae *(2.26 Mb versus 1.83 Mb), it has 37% fewer instances of the USS motif, and fewer high-confidence terminators (17%) contain the motif. Interestingly, the most common motif within high-confidence terminators in *H. ducreyi *(AAGCGGT) matches the USS for the other Pasteurellaceae (AAGTGCGGT) except for an excision of a GT dinucleotide sequence. This shorter motif occurs 1,371 times in the genome (expected frequency is 140 occurrences), suggesting that it may also function as a USS. Previous studies [10,23] failed to find any USS in *H. ducreyi *due to this difference from the expected *H. influenzae*-derived motif. No previous study has reported on the uptake signal sequences for *Mannheimia succiniciproducens*. Thus, in addition to quantifying their involvement in intrinsic terminators, we report the first evidence of these signals in these species.

In *N. meningitides Z2491*, 81% of the USS instances within intergenic regions that have a high-confidence terminator are contained within a high-confidence terminator (though perhaps not the best one). Percentages are similar in the other *Neisseria*. In *H. influenzae*, on the other hand, only 34% of such USS sites are contained within a terminator. The other Pasteurellacaea have similar low percentages (29% to 42%).

#### Extended USS motifs

**Figure 4:**
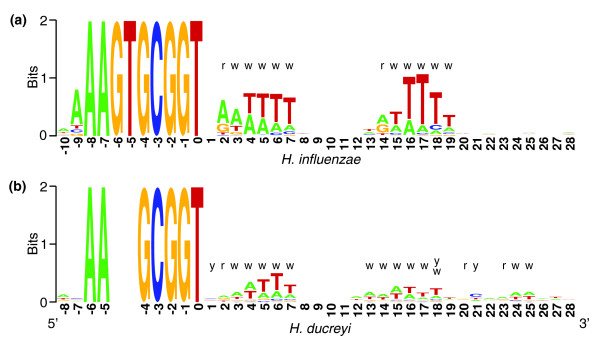
Extended motif for *H. ducreyi *and *H. influenzae*. **(a) **A sequence logo [26,27] created from the regions surrounding occurrences of USS motifs in *H. influenzae*. Position +1 is the first position following the USS motif. The previously reported extended motif is shown above the letters. **(b) **A sequence logo created from the regions surrounding occurrences of the conjectured USS motif in *H. ducreyi*. A gap of two nucleotides has been introduced to align the *H. ducreyi *motif with the *H. influenzae *motif. (An alternative alignment places position -4 in *H. ducreyi *across from position -6 in *H. influenzae*.) Examining the frequencies, we can derive a consensus pattern as follows. We mark a position with a 'w' if more than 70% of the occurrences contain an A or a T (expected frequency of an A or T = 60%). We mark a column with a 'y' or 'r' if more than 60% of the occurrences have a T or a C (for 'y') or A or a G (for 'r'). The expected frequency for either case is 50%. The 'rwwwwnnnn' from positions +2 to +11 matches the previously identified extended motif for *H. influenzae*, though the rwwww motif is not as clear. The subsequent 'nnrwwwww' from the extended *H. influenzae *motif is not exactly matched in *H. ducreyi *but there is a general bias toward A and T residues extending to about +25.

The USS sites in both *Neisseria *and Pasteurellaceae are often found within a longer, imperfectly conserved motif [10,11]. In *H. influenzae*, the extended motif follows the pattern: aaAAGTGCGGTnrwwwwwnnnnnnrwwwww, where 'n' indicates any base, 'r' indicates {A,G} and 'w' indicates {A,T} (Figure 4a). In *Neisseria*, the extended motif is aaatGCCGTCTGAAa. In *H. ducreyi*, we find that the extended motif is AAGCGGTyrwwwwwnnnn followed by an overrepresentation of A and T residues until about 25 nt following the 3' end of the core motif (Figure 4b), but this pattern is more weakly conserved. In all cases, these extended motifs begin with a stretch of adenine residues, which, if a pair of USS motifs is arranged into a hairpin in the +- configuration, will cause the hairpin to be flanked by adenines on the 5' end and thymines on the 3' end. Because the extended motifs are apparent even among USSs that are not involved in hairpins, we cannot directly conclude that because such a USS hairpin is followed by a stretch of thymines, it is a terminator. However, a more extensive analysis does support the conclusion that USS hairpins are maintained to promote transcription termination.

#### Evidence that paired USS motifs function as terminators

We searched the genome of *N. meningitidis Z2491 *for closely spaced (within 10 nt), oppositely directed instances of USS sites. Such hairpin configurations of the USS sites occur most frequently between oppositely oriented genes (tail-to-tail regions, → ←, Table 4), and considering both the relatively small number of tail-to-tail regions and their short total length, these regions are enriched for hairpin instances. This remains true even accounting for the varied distribution of single USS motifs within each region type ('No. of USS' column in Table 4). The conservation of the extended motif on either side of the hairpin also supports the conclusion that these function as terminators. Looking at the reference strand as recorded in GenBank, we see that the conservation of T and A nucleotides flanking these hairpins follows the pattern expected given the region in which the hairpin is found (Table 4). For example, those between two forward-directed genes (forward tail-to-head regions, → →) have more conserved Ts in their T-tail region on average, while for reverse tail-to-head regions (← ←) the opposite is true and more nucleotides are conserved in the A-tail. Both the A-tail and T-tail are enriched for their respective nucleotides for hairpins in tail-to-tail regions. The patterns are similar for the other organisms listed in Table 3.

While overall there are about equal numbers of occurrences of a USS motif and its reverse complement in all the genomes in Table 5, there is a strong bias in the orientations of the USS motifs located near each other in the *Neisseria *genus and a similar, less pronounced bias in the Pasteurellacaea. This bias was previously noted in *H. influenzae *[10] and *N. meningitidis *[11], and we observe it in *N. gonorrhoeae *and other Pasteurellacaea. In *Neisseria*, the configuration GCCGTCTGAA closely followed by TTCAGACGGC (+- in Table 5) is far more common than TTCAGACGGC closely followed by GCCGTCTGAA (-+ in Table 5) despite both arrangements forming high-quality hairpins. This is likely because the +- configuration places the AAAT at the 5' end of the extended motif into positions to form the A- and T-tails of the terminator. This is also true in the Pasteurellacaea, in which the +- configuration places the AAAA sequence at the 5' end of the extended motif into the tail positions. The situation in these organisms is more complex, however, as the +- configuration also causes the long 3' ends of the extended USS motifs to interact. This interference of the 3' ends of the extended motifs may be the cause of the reduced bias in the Pasteurellacaea and perhaps the lessened prevalence of the USS motif within terminators. As expected, if the primary reason for nearby USS motifs is development of terminator hairpins, consecutive instances of the motif in the same orientation within 10 nt of each other are rare (++ and -- in Table 5).

**Figure 5:**
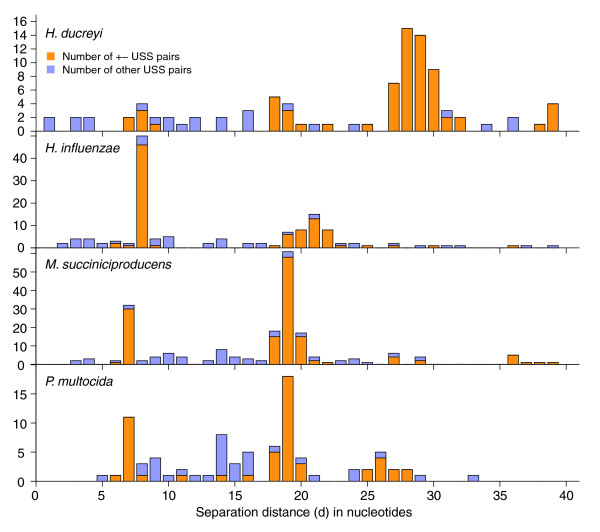
Bias toward +- configuration by separation distance. For each of four Pasteurellacaea, the height of the bar gives the total number of paired USS motifs separated by the given distance. The orange (lower) portion of the bar gives the number of pairs in the +- configuration. In *H. influenzae*, local peaks around approximately 8 and approximately 19-22 are due to the preservation of the extended USS motif. The distribution of distances is different for *H. ducreyi*, which has a peak of pairs (all in the +- configuration) at separations of 27 to 30, and the preference for the +- motif is not apparent until larger separation distances.

The number of the pairs separated by a given distance *d *is plotted in Figure 5, and the proportion of those pairs in the +- configuration is indicated. Spikes at separations of approximately 8 nt and approximately 19-22 nt reflect the best arrangements to preserve the long 3' end of the extended USS motif [11]. After a separation of about 30 nt, there is no bias toward +- in any of the Pasteurellacaea. In *H. ducreyi*, 75% of the 83 USS pairs spaced approximately 30 nt apart are in the +- configuration. However, at distances approximately 25 nt a majority of the pairs are in the -+ arrangement. One possible explanation for this is that the shorter *H. ducreyi *core motif is more dependent on the preservation of the full extended motif, which itself is biased toward A and T residues over a longer region. That hairpin USS pairs in *H. ducreyi *are forced either to be separated by a long loop or to overlay the GC-rich core motif with the AT-rich extended motif may account for the lower percentage of terminators that contain USS motifs in this organism.

The bias in genomic context, conservation pattern of the extended motifs, and the preference for an orientation that creates good A- and T-tails support the notion that USS hairpin configurations are maintained to promote transcription termination, and Table 3 quantifies the propensity for terminators to include USS signals. Evolutionary expediency may be the reason for the co-location of these signals. The prevalence of so many copies of a short motif and its complement (as required to ensure selective uptake) likely facilitates the creation of many hairpins that can be co-opted for termination simply by point mutations to create a strong T-tail. By reusing the USS, it is no longer necessary for there to be a series of coordinated, complementary mutations for the development of hairpin structures.

## Conclusion

We have described a highly efficient, accurate computational system for predicting Rho-independent transcription termination in bacterial genomes, and we have used this system to predict terminators in hundreds of genomes for which no such predictions were previously available. Our predictions for 343 organisms are available on the web [18], and can be downloaded in bulk, by organism, or they can be searched based on score, genomic context, and features of their sequence. Terminators downstream of specified genes can be found as well. They represent the most complete set of terminator predictions yet available.

The new system is over 10 times faster than an earlier algorithm by one of the authors and thousands of times faster than a recently described system [3], with comparable sensitivity and specificity.

The speed and accuracy of TransTermHP has facilitated the discovery of a likely DNA uptake motif in *H. ducreyi *where none was previously known. Though most USS signals are not involved in termination, we have given more evidence that some USS pairs within *Neisseria *and Pasteurellacaea are maintained in hairpin configurations in order to affect transcription termination, and we have quantified the tendency for terminators to include USS motifs.

TransTermHP is designed to detect the common, classic intrinsic terminator motif: a hairpin stem followed by a poly-U tail. The method may be less accurate in detecting those terminators that deviate from this motif, for example, by lacking the uracil-rich tail. Given the high sensitive and specificity of TransTermHP, if it is unable to find many high-quality terminators in an organism that lacks a Rho homolog, this may be a hint that some variant of the intrinsic terminator motif is used.

In addition to its immediate utility for accurately finding Rho-independent terminators, we hope the method and accompanying software will be a useful starting point for developing other improved systems for terminator prediction, investigations of structural properties of intrinsic terminators (in the spirit of [5]), as well as related problems such as discovery of anti-termination structures [24].

## Materials and methods

Genomic data for the 343 organisms was downloaded on 6 June 2006 from [25]. Annotations were taken from the .ptt file accompanying the genomic sequence. Accession and version numbers for the sequences used are available as Additional data file 3. TransTermHP was run using the default search parameters and the -p scoring parameter to produce predictions for the best terminator following each gene in these organisms (available on the web [18], --bag option to TransTermHP) as well as the best terminators in each robust tail-to-tail region (Additional data file 2, --t2t-perf option to TransTermHP). The former were used when comparing performance to [3], while the latter was used to assess the importance of Rho-independent termination within an organism. Experiments were run on a 3.2 GHz Intel Xeon processor running Linux. High-confidence terminators are those with scores ≥ 76.

The C++ source code for TransTermHP is available (Additional data file 1) under an open source license.

## Additional data files

The following additional data are available with the online version of this paper. Additional data file 1 is the complete C++ source code for the TransTermHP system. Additional data file 2 is a zipped archive of plain text files listing the highest-scoring terminators in robust tail-to-tail regions for each of the 343 genomes studied. As described above, the entire robust tail-to-tail region is searched; if the region is less than 500 nt, then the 500 nt downstream of the stop codon is searched (stopping if a gene start is encountered). In the case of ties, all terminators with the high score are output. Additional data file 3 is a plain text file listing, for each genome, the accession and version numbers for the sequences used. Additional data file 4 is a zipped archive of text files listing the best terminators found downstream of each of the genes of the 343 organisms studied.

## Supplementary Material

Additional data file 1 Complete C++ source code for the TransTermHP system. Click here for file

Additional data file 2 As described in the text, the entire robust tail-to-tail region is searched; if the region is less than 500 nt, then the 500 nt downstream of the stop codon is searched (stopping if a gene start is encountered). In the case of ties, all terminators with the high score are output. Click here for file

Additional data file 3 Accession and version numbers for the sequences of each genome used. Click here for file

Additional data file 4 Best terminators found downstream of each of the genes of the 343 organisms studied. Click here for file

## Figures and Tables

**Table 1:** Parameters used to evaluate hairpins

Pairing	Energy
G-C	-2.3
A-T	-0.9
G-T	1.3
Mismatch	3.5
Gap	6.0
Loop_pen(*n*)	1·(*n *- 2)

Parameters used to evaluate the energy of a potential hairpin where *n *is the length of the hairpin loop.

**Table 2:** Bacteria outside of the Firmicutes with many high-confidence terminators

Organism	*F*_*TT*_	*N*_*TT*_
*Neisseria *(β-proteobacteria)		
*Neisseria meningitidis *Z2491	79	360
*Neisseria meningitidis *MC58	77	357
*Neisseria gonorrhoeae *FA 1090	77	356
*Vibrio *(γ-proteobacteria)		
*Vibrio fischeri *ES114	76	714
*Vibrio parahaemolyticus*	74	955
*Vibrio vulnificus *CMCP6	72	866
*Vibrio vulnificus *YJ016	65	919
*Vibrio cholerae*	64	739
Pasteurellaceae (γ-proteobacteria)		
*Pasteurella multocida*	80	360
*Haemophilus influenzae *86 028NP	75	322
*Haemophilus influenzae*	76	292
*Mannheimia succiniciproducens *MBEL55E	71	445
*Haemophilus ducreyi *35000HP	63	310
Other γ-proteobacteria		
*Psychrobacter cryohalolentis *K5	69	485
*Psychrobacter arcticum *273-4	65	417
*Pseudoalteromonas haloplanktis *TAC125	64	667
δ-Proteobacteria		
*Desulfovibrio desulfuricans *G20	62	661
Fusobacteria		
*Fusobacterium nucleatum*	66	267

Bacteria from outside the Firmicutes division for which TransTermHP finds a high-confidence terminator following > 60% of the genes that were followed by at least two convergently transcribed genes (that is, robust tail-to-tail regions). A tail-to-tail region extends from 25 nt upstream of the stop codon to 500 nt downstream of it, or until the stop codon of the convergently transcribed gene is found, whichever is longer. The region between two convergently transcribed genes counts as two robust tail-to-tail regions (one for each strand) if both genes are preceded by a co-directed gene. *F*_*TT *_is the percentage of robust tail-to-tail regions containing a terminator, and *N*_*TT *_is the number of robust tail-to-tail regions in each organism.

**Table 3:** USS motifs in the best high-confidence terminators following genes

Organism	USS	*N*	*R*	*G *(%)	*U*	*P *(%)
*Neisseria*						
*N. meningitidis *Z2491	5'-GCCGTCTGAA-3'	1,892	827	40	469	57
*N. meningitidis *MC58	5'-GCCGTCTGAA-3'	1,935	801	39	444	55
*N. gonorrhoeae *FA 1090	5'-GCCGTCTGAA-3'	1,965	828	41	451	54
Pasteurellaceae						
*H. influenzae*	5'-AAGTGCGGT-3'	1,471	644	39	148	23
*H. influenzae *86 028NP	5'-AAGTGCGGT-3'	1,516	668	37	147	22
*P. multocida*	5'-AAGTGCGGT-3'	927	796	40	133	17
*M. succiniciproducens *MBEL55E	5'-AAGTGCGGT-3'	1,485	848	36	248	29
*H. ducreyi *35000HP	5'-AAGCGGT-3'	1,371	530	31	84	16

Column *N *gives number of USS instances in the genome. Column *R *gives the number of genes followed by a high-confidence terminator, and column *G *gives this as a percentage of the total number of genes. These genes are the most likely operon ends. Column *U *gives the number of these likely operon ends for which the best high-confidence terminator contains at least one exact match to the USS motif or its reverse complement. Column *P *gives this number as a percentage of the likely operon ends (*P *= *U*/*G*). (Because of the requirements for high-confidence terminators, the USS motif likely pairs with a similar, but perhaps imperfect, USS motif on the opposite side of the hairpin stem.) A large fraction of predicted operon ends have a terminator that contains a USS signal. For *H. ducreyi *the motif AAGCGGT is not known to be a USS; however, its prevalence in the genome and similarity to the USS motif in other Pasteurellaceae lead us to conjecture that it functions as a USS. Only 1% of the likely operon ends in *H. ducreyi *had a terminator containing the USS motif found in the other Pasteurellaceae.

**Table 4:** Analysis of all USS hairpins in *N. meningitidis Z2491*

				Average no. of A/Ts	
				
Region	No. of reg	No. of USS	No. of hairpins	A-tail	T-tail
Tail-to-tail	268	375	94	3.33	3.18
Reverse tail-to-head	761	396	61	2.75	1.97
Forward tail-to-head	652	364	63	2.08	3.05
Intragenic	2,065	635	3	3.67	3.33
Head-to-head	270	104	10	2.40	1.70

Analysis of all hairpins of the GCCGTCTGAA...TTCAGACGGC and TTCAGACGGC...GCCGTCTGAA motif in *N. meningitidis Z2491*. Hairpins were found by exhaustive search as described in the text. The pattern of conservation of nucleotides in the regions flanking the hairpins reflects that expected if the hairpins are being maintained to function as transcription terminators. The 'No. of reg' column gives the number of intergenic regions of positive length of each type. The 'No. of USS' column gives the number of USS instances (not necessarily paired) within each region type. The 'No. of hairpins' column lists the number of hairpin configurations of USS motifs (separated by ≤ 10 nt) that overlap each type of region. (For intragenic regions, we require that the hairpin be wholly contained within a gene.) The 'A-tail' column gives the average number of *A *residues in the five bases preceding the first occurrence of the motif in the pair. The 'T-tail' column gives the average number of *T *residues in the five bases following to the second occurrence of the motif in the pair.

**Table 5:** Orientation of USS motifs and closely spaced USS pairs

Organism	Total +	Total -	++	--	+-	-+
*Neisseria*						
*N. meningitidis *Z2491	958	934	0	0	226	5
*N. meningitides *MC58	966	969	0	0	229	4
*N. gonorrhoeae *FA 1090	1,011	954	1	1	215	5
Pasteurellaceae						
*H. influenzae*	737	734	1	0	50	25
*H. influenzae *86 028NP	734	782	0	0	52	23
*P. multocida*	468	459	0	0	13	8
*M. succiniciproducens *MBEL55E	730	755	0	0	31	20
*H. ducreyi *35000HP	717	654	0	2	6	8

For the organisms listed in the table, the USS motif occurs with approximate equal frequency on each strand. The 'Total +' and 'Total -' columns give the number of times the motif occurs on the reference strand as deposited in GenBank (+) and how many times it occurs on the complementary strand (-). The '++' and '--' columns count the number of occurrences in which the motifs occurred on the same strand within 10 nt of each other. As expected, since these configurations do not induce hairpins, they are relatively rare. The '+-' and '-+' columns give the number of occurrences of the USS motifs on opposing strands separated by ≤ 10 nt. There is an overabundance of +- pairs, representing, for example, a preference for the GCCGTCTGAA...TTCAGACGGC hairpin over the TTCAGACGGC...GCCGTCTGAA hairpin.
